# *Aphaereta ceratitivora* sp. n. (Hymenoptera, Braconidae), a new parasitoid of *Ceratitis capitata* (Wiedemann) (Diptera, Tephritidae) from the Azores

**DOI:** 10.3897/zookeys.222.3618

**Published:** 2012-09-18

**Authors:** Kees van Achterberg, Tânia Teixeira, Luísa Oliveira

**Affiliations:** 1 Dept. Terrestrial Zoology, NCB Naturalis, Postbus 9517, 2300 RA Leiden, The Netherlands; 2 Departamento de Biologia, Universidade dos Açores, Rua da Mãe de Deus, 9501-801 Ponta Delgada, S. Miguel, Açores, Portugal; 3 IBB-CBA, CIRN Departamento de Biologia, Universidade dos Açores, Rua da Mãe de Deus, 9501-801 Ponta Delgada, S. Miguel, Açores, Portugal

**Keywords:** *Aphaereta ceratitivora*, *Ceratitis capitata*, new species, Azores, gregarious parasitoid

## Abstract

A new gregarious larval-pupal endoparasitoid of *Ceratitis capitata* (Wiedemann) (Diptera: Tephritidae) is described and illustrated: *Aphaereta ceratitivora* **sp. n.** (Braconidae: Alysiinae: Alysiini).

## Introduction

In recent years interest in the parasitoid Hymenoptera has grown as a result of the increasing demand for biological methods for pest control and their possible use as natural enemies. The Braconidae are the second largest family of this order, the majority of species are primary parasitoids of immature stages of Lepidoptera, Coleoptera and Diptera (Sharkey 1993).

*Ceratitis capitata* (Wiedemann, 1824) (Diptera: Tephritidae) or Mediterranean fruit fly (Medfly) is a pest that causes substantial economic losses in the Mediterranean fruit production due to their high dispersal ability and ecological plasticity (Liquido et al. 1991; Gillani et al. 2002). In Azores (Portugal) a survey was done to see if there were possible candidates for the control of *Ceratitis capitata*, before considering the introduction of exotic species, that can cause adverse effects on the native parasitoids and non-target species (Oliveira et al. 2008).

From two parasitized pupae of *Ceratitis capitata* collected in São Miguel Island (Azores, Portugal) emerged in total 12 adult parasitoids, belonging to a new species of the genus *Aphaereta* Foerster, 1862 (Alysiinae: Alysiini). Six adult parasitoids emerged per pupa; rearing in the lab resulted in four parasitoids per host pupa at 20°C (the optimum temperature for development). The subfamily Alysiinae, with 2321 catalogued species worldwide (Yu et al. 2012) has a prominent position within the Braconidae family (van Achterberg 1993, Dolphin and Quicke 2001) and consists of the tribes Alysiini and Dacnusini. The species of this subfamily are endoparasitoids of dipterous larvae, with oviposition into the egg or the larva of the host and emergence from the host puparium. Wharton (2002) used this character, along with the possession of exodont mandibles, to define the subfamily. Almost all Dacnusini are parasitoids of leaf- and stem-mining dipterans, usually Agromyzidae (Wharton 2002), but Alysiini attacks a wide range of dipterous hosts from at least 20 different families (Wharton 1980).

## Description

### Aphaereta (Aphaereta) ceratitivora

van Achterberg & Oliveira sp. n.

urn:lsid:zoobank.org:act:6AA4B4C3-1AD2-4453-B9F3-E63425D9302A

http://species-id.net/wiki/Aphaereta_ceratitivora

#### Type material.

Holotype, ♀ (RMNH), “Portugal: Azores, Ponta Delgada, reared in lab., summer 2010, L. Oliviera, RMNH’11”, “ex *Ceratitis capitata* (Wied.)”. Paratypes: 19 ♀ + 20 ♂ (RMNH), with same label data; 10 ♀ + 3 ♂ (RMNH), “Portugal: Azores, S. Miguel Isl., Vila Franca, 15.x.2008, [reared] in lab. ex pupae of *Ceratitis capitata* (Tephrit.) coll. from *Capsicum annuum*, L. Oliviera, RMNH’09”.

#### Diagnosis.

Antenna of ♀ with 18–20 segments and 1.1–1.2 times as long as fore wing; pedicellus yellowish-brown, not contrasting with scapus; third antennal segment of ♀ dark brown or brown basally and comparatively slender (Fig. 6); outer side of fourth antennal segment of ♀ straight or nearly so (Fig. 6); fourth-seventh antennal segments of ♀ moderately shiny and dark brown; ventral convex area of side of pronotum moderately narrow and yellowish-brown; medio-posterior depression of mesoscutum absent; wing membrane slightly infuscate; axillar depression narrow to medium-sized and smooth; tegulae brown or dark brown, darker than fore femur; ventrally hind basitarsus narrowly acutely protruding postero-ventrally; hind tibia distinctly setose baso-dorsally; hind basitarsus moderately slender and often more or less infuscate; tarsal claws narrow (Fig. 7); median carina of propodeum in lateral view hardly protruding and narrowly lamelliform (Fig. 12); first metasomal tergite strongly widened posteriorly, medially densely and finely rugulose and dark or pale brown (Fig. 11); setose part of ovipositor sheath 0.6 times as long as metasoma and 0.7–0.8 times as long as hind tibia; length of fore wing 1.6–2.4 mm and of body 1.6-2.2 mm.

**Figures 1–13.**
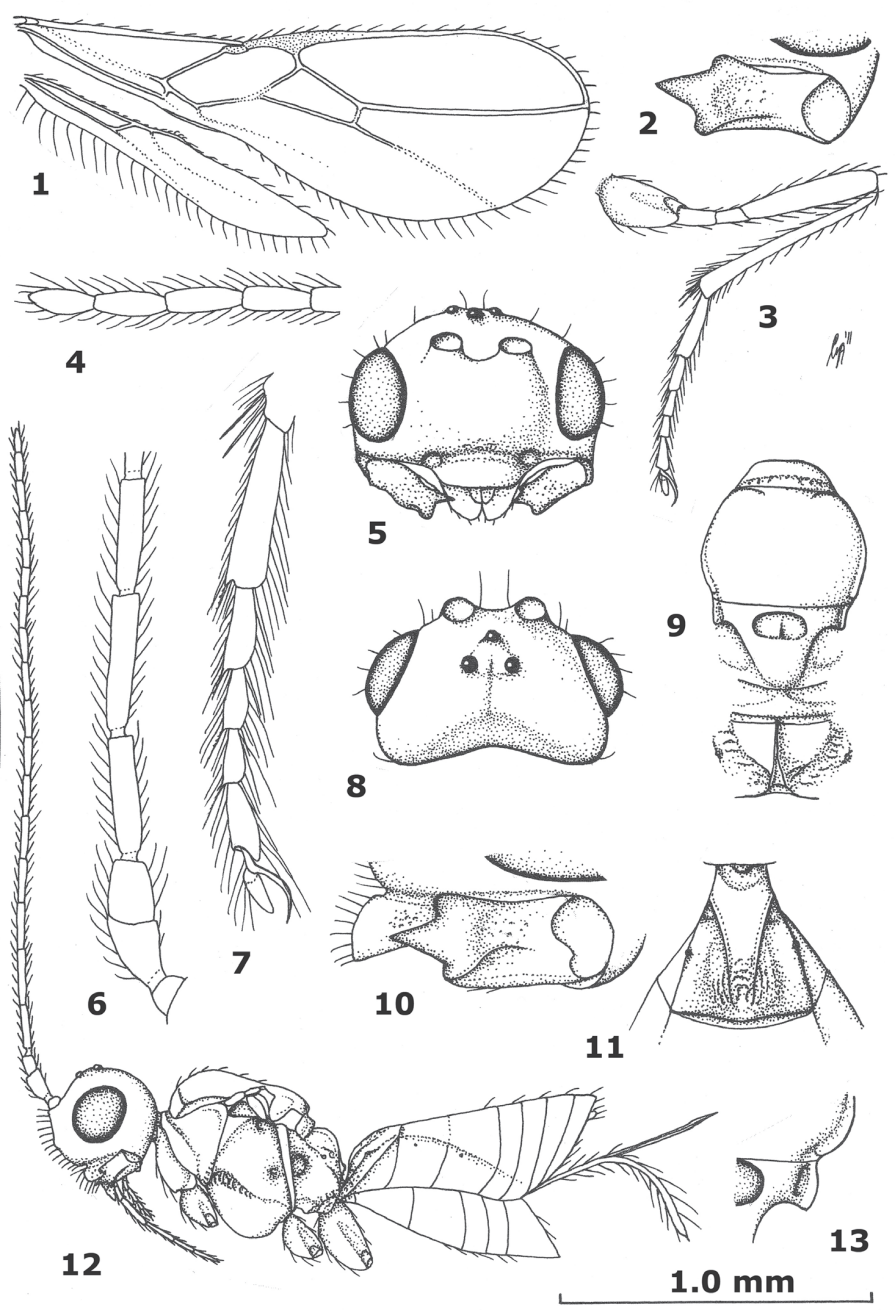
*Aphaereta ceratitivora* sp. n., holotype, ♀. **1** wings **2** mandible full view of third tooth **3** hind leg **4** apical segments of antenna **5** head anterior **6** basal segments of antenna **7** hind tarsus **8** head dorsal **9** mesosoma dorsal **10** mandible full view of first tooth **11** first metasomal tergite dorsal **12** habitus lateral **13** axillar depression.

#### Description.

Holotype, ♀, length of body 1.7 mm, of fore wing 1.8 mm.

*Head*. Antenna 1.2 times length of fore wing and 1.6 times body (Fig. 12), with 19 segments, setae erect and about 1.3 times as long as width of segment, length of third segment 0.8 times fourth segment (Fig. 6), length of third, fourth and penultimate segments 4.0, 5.0 and 2.6 times their width, respectively; apex of scapus oblique and pedicellus medium-sized (Fig. 6); maxillary and labial palp with 6 and 4 segments, respectively; length of maxillary palp equal to height of head; Ocular Ocellar Line : diameter of posterior ocellus : Posterior Ocellar Line = 8:3:4; frons glabrous and smooth, nearly flat; length of eye in dorsal view 1.7 times the temple (Fig. 8); eyes glabrous except for a few setae; temples parallel-sided behind eyes (Fig. 8); median groove of vertex shallow; face smooth, but medio-ventrally punctulate and somewhat rugulose, with long erect setae; clypeus largely smooth (but somewhat punctate laterally), flattened medially, long, setose, not tuberculate laterally and ventral rim truncate (Fig. 5); epistomal groove rather wide, laterally smooth and distinct (Figs 10, 12); anterior tentorial pits medium-sized (Fig. 5); length of malar space 0.2 times basal width of mandible; mandible largely smooth, hardly widened subapically, its medial length 1.75 times maximum width, second tooth much longer than both other lobe-shaped teeth, without incision between first and second tooth, third tooth with curved carina (Figs 2, 10); head 1.6 times as wide as mesoscutum (Figs 5, 9).

*Mesosoma*. Length of mesosoma 1.3 times its height; pronope absent, but with a transverse crenulate groove and no antescutal depression; side of pronotum smooth, antero-medially and ventro-posteriorly finely crenulate (Fig. 12), ventral area slightly convex, medium-sized and yellowish-brown; precoxal sulcus only anteriorly and medially moderately crenulate (Fig. 12); mesosternal sulcus wide and coarsely crenulate; metapleuron smooth dorsally and medially, crenulate-rugose ventrally; notauli absent, only anteriorly with horizontal carina (Fig. 9); medio-posterior depression of mesoscutum absent; mesoscutum glabrous (except for a few setae), smooth and with complete lateral carina; scutellar sulcus wide, semi-circular and deep, with one short crenula; axillar depression narrow and rectangular, smooth and rather shallow (Fig. 13); scutellum slightly convex; anteriorly metanotum without median carina and not protruding dorsally (Fig. 12); surface of propodeum mainly smooth dorsally and remainder rather sparsely rugulose (Fig. 9), without lateral protuberance, its median carina moderately protuberant and with narrow triangular medial area (Fig. 9).

*Wings*. Fore wing: 1-SR normal; 2-SR oblique; r:3-SR:SR1 = 3:16:51; r somewhat widened (about 3 times as long as its own width), 0.7 times width of pterostigma and issued just before middle of pterostigma (Fig. 1); pterostigma narrow, elliptical (Fig. 1); SR1 straight and ending at apex of wing (Fig. 1); 1-CU1:2-CU1 = 1:6; 2-SR:3-SR: r-m = 19:32:8; first subdiscal cell open ventrally and apically; CU1a just below level of 2-CU1; m-cu converging to 1-M. Hind wing: no trace of cu-a and m-cu (Fig. 1), others veins completely sclerotized.

*Legs*. Hind coxa smooth; tarsal claws very slender (Fig. 7), no distinct protuberance but widened basally; length of femur, tibia and basitarsus of hind leg 5.0, 11.0, and 4.3 times their width, respectively; hind femur largely smooth and parallel-sided; hind tibia somewhat widened apically and subbasally setose; hind tarsal segments with a short and sparsely setose ventro-apical protuberance (Fig. 7); hind basitarsus comparatively robust and slightly narrowed basally (Fig. 7); length of hind tibial spurs 0.4 and 0.5 times hind basitarsus.

*Metasoma*. Length of first tergite equal to its apical width, its surface distinctly convex and finely rugose medially, remainder largely smooth (Fig. 11), its dorsal carinae distinct in basal 0.8 and tergite widened behind spiracles (Fig. 11); dorsope rather large; second tergite smooth; ovipositor straight; length of setose part of ovipositor sheath 0.27 times fore wing, 0.6 times metasoma and 0.7 times hind tibia, with long setae, ribbon-shaped (except apically) and with a short apical spine (Fig. 12); hypopygium medium-sized and apically subtruncate (Fig. 12).

*Colour*. Black or dark brown; scapus and pedicellus of antenna, mandible (but margins darkened), metasoma largely ventrally and legs (but telotarsi, apex of hind tibia and hind basitarsus slightly infuscate) yellowish-brown; palpi pale yellowish; propleuron, pronotal side ventrally, tegulae, first tergite and veins brown; pterostigma (but pale apically), parastigma and remainder of metasoma rather dark brown; wing membrane slightly infuscate.

*Variation*. Antenna of ♀ with 18 (4), 19 (17) or 20 (7) segments (but one female with 20 segments in left antenna has 21 segments in right antenna) and 1.1–1.2 times as long as fore wing, of ♂ with 19 (1), 20 (3), 21 (8), 22 (5) or 23 (3) segments and 1.2–1.5 times as long as fore wing; length of fore wing 1.6–2.4 mm, and of body 1.6–2.2 mm; first metasomal tergite 1.0–1.2 times longer than its apical width; length of setose part of ovipositor sheath 0.27–0.32 times fore wing and 0.7–0.8 times hind tibia; axillar depression narrow to medium-sized, rectangular to narrow triangular; tegulae brown or dark brown, darker than fore femur; third antennal segment dark brown or largely brown and rest dark brown; hind tarsus often entirely dark brown or infuscate; second submarginal cell of fore wing parallel-sided or widened basally.

#### Biology.

Gregarious parasitoid of the Mediterranean fruit fly, *Ceratitis capitata* (Wiedemann, 1824) (Tephritidae).

#### Distribution.

Portugal (Azores); most likely also France, but no material available for study (see below).

#### Etymology.

From the generic name of its host (*Ceratites*) and “voro” (Latin for “devour”), because it is devouring this host.

#### Notes.

Similar to the gregarious Nearctic *Aphaereta pallipes* (Say, 1829), but this species is a parasitoid of other families and has the setose part of the ovipositor sheath about as long as the metasoma and longer than the hind tibia. The Mediterranean fruit fly has been reported as host of *Aphaereta minuta* (Nees, 1911) from South France (Ghesquière 1950, Martin 1952, Narayanan and Chawla 1962), but this is most likely a misidentification. *Aphaereta minuta* is very similar, but differs in having the hind basitarsus slenderer (Fig. 21) (less so in *Aphaereta ceratitivora*; Fig. 7), the antennal segments of ♀ up to 22 (up to 20 segments), the axillar depression wide and finely crenulate (comparatively narrow and smooth), clypeus low basally (steeply elevated), the first tergite less widened posteriorly (more widened posteriorly; Fig. 11)), the tegulae and fore femur similarly coloured (tegulae darker than fore femur) and gregarious parasitoid of dipterous larvae in dung and rotting organic matter; e.g., *Scatophaga* species in rotting seaweed, and *Sarcophaga* species in human excrements and rotting *Sepia* species (gregarious parasitoid of *Ceratitis capitata* in fruits).

**Figures 14–25.**
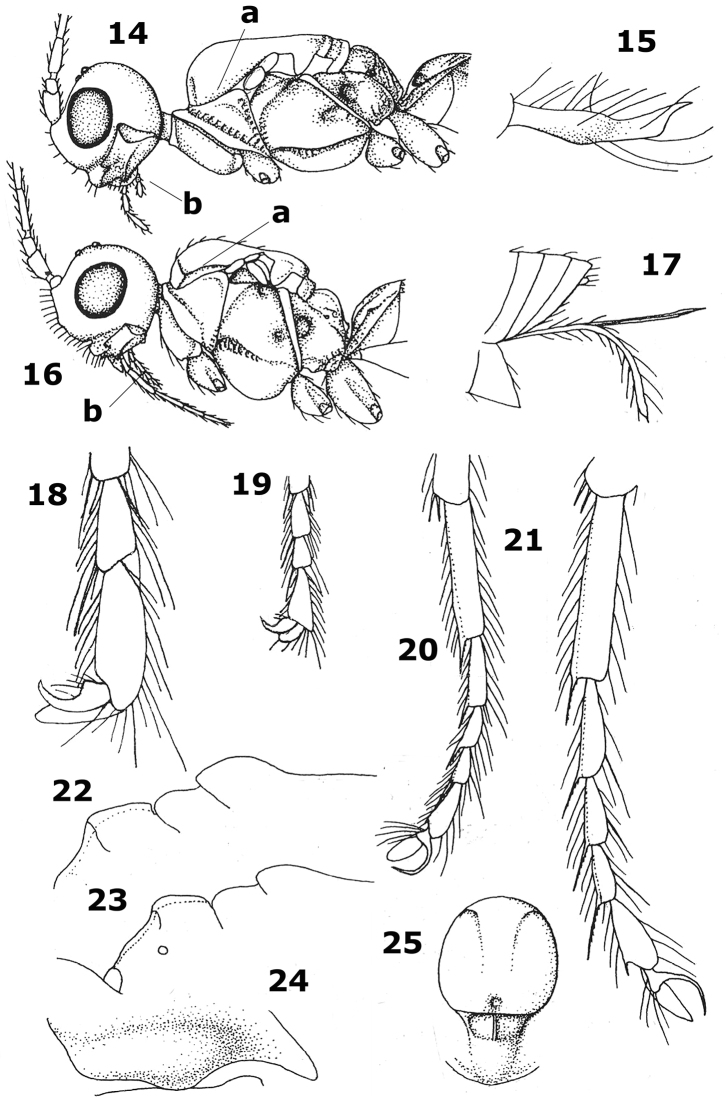
*Aphaereta* spp. **14, 16** head and mesosoma lateral **15, 17** ovipositor sheath **18, 19** hind tarsal claw **20, 21** hind tarsus **22, 23** scutellum, metanotum and propodeum lateral **24** convex ventral part of pronotal side **25** mesothorax dorsal.

### Key to main groups of the genus *Aphaereta* Foerster in Europe

**Table:** 

1	Lateral carina of mesoscutum absent in front of tegulae (Fig. 14a); ovipositor sheath very aberrantly shaped, widened submedially and up curved apically (Fig. 15); labial palp with 2 segments (Fig. 14b) and maxillary palp with 4–5 segments	*Aphaereta falciger* Graham, 1960
–	Lateral carina of mesoscutum present in front of tegulae (Fig. 16a); shape of ovipositor sheaths normal, parallel-sided and straight (Fig. 17); labial palp with 3–4 segments (fig. 16b) and maxillary palp with 5–6 segments	2
2	Tarsal claws comparatively robust, gradually widened submedially (Figs 18, 19) and third antennal segment of female about 3 times as long as wide	*Aphaereta stigmaticalis* group
–	Tarsal claws very slender, hardly widened submedially (Figs 20, 21); third antennal segment of female usually 4–7 times as long as wide	3
3	Medio-posterior depression of mesoscutum absent (Fig. 9); setose part of ovipositor sheath 0.8–1.1 times as long as hind tibia; *Aphaereta minuta* group	4
–	Medio-posterior depression of mesoscutum present (Fig. 25), but sometimes minute; setose part of ovipositor sheath 0.3–1.5 times as long as hind tibia	*Aphaereta tenuicornis* group
4	Scutellum distinctly convex medially (Fig. 22; hind femur erect bristly setose and comparatively widened apically in lateral view	*Aphaereta difficilis* Nixon, 1939
–	Scutellum slightly convex medially (Fig. 23); hind femur less bristly setose and more slender in lateral view (Fig. 3)	5
5	Tegulae brown or dark brown, darker than fore femur; hind basitarsus less slender (Fig. 7; about 4.5 times as long as its maximal width); axillar depression narrow to medium-sized and smooth (Fig. 13); ventral convex area of side of pronotum comparatively narrow (Fig. 12); antenna of ♀ with 18–20 segments; gregarious parasitoid of Tephritidae; Azores, and probably southern France	*Aphaereta ceratitivora* sp. n.
	Note. Similar to the Nearctic *Aphaereta pallipes* (Say, 1829), but this species is a gregarious parasitoid of other Diptera families and has setose part of ovipositor sheath about as long as metasoma and longer than hind tibia.
–	Tegulae yellowish-brown, similar to colour of fore femur; hind basitarsus comparatively slender (Figs 20, 21; about 5 times as long as its maximum width); axillar depression large triangular; ventral convex area of side of pronotum comparatively wide (Fig. 24); antenna of ♀ with 19–22 segments; parasitoid of Scatophagidae (*Scatophaga* sp. in rotting seaweed), and Sarcophagidae (*Sarcophaga* sp. in human excrements and rotting *Sepia*)	*Aphaereta minuta* (Nees, 1811)

## Supplementary Material

XML Treatment for Aphaereta (Aphaereta) ceratitivora
